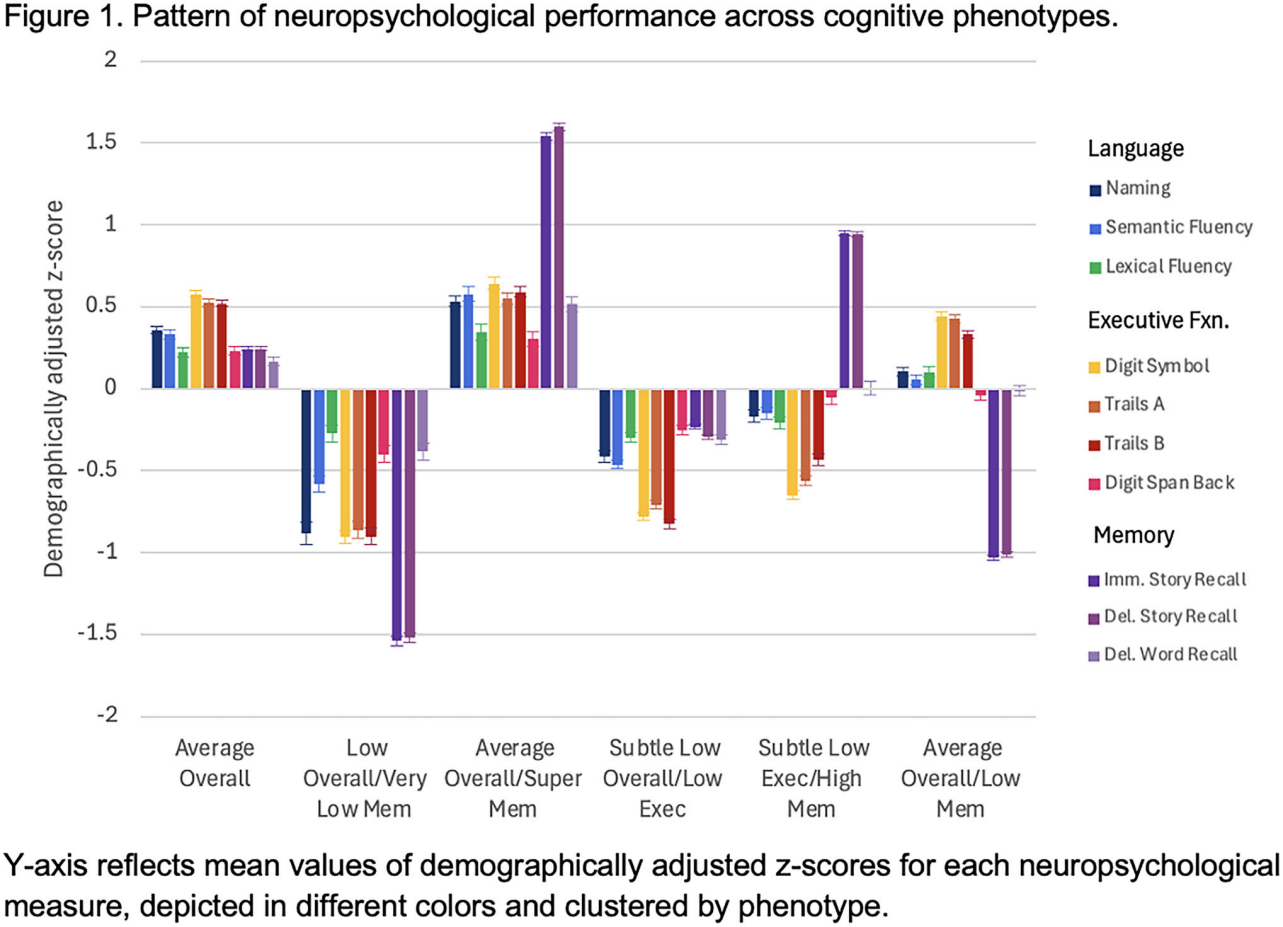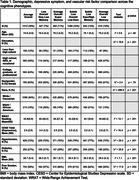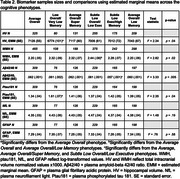# Cognitive heterogeneity of data‐driven phenotypes and associations with ADRD and cerebrovascular biomarkers in the Atherosclerosis Risk in Communities Study

**DOI:** 10.1002/alz70857_104395

**Published:** 2025-12-25

**Authors:** Alexandra J. Weigand, Fareshte Erani, Caitlin M. Terao, Alden L. Gross, Keenan A. Walker, Emily C. Edmonds, Katherine J. Bangen, Mark W. Bondi, B Gwen Windham, James R Pike, David S. Knopman, Rebecca F. Gottesman, Priya Palta, Kelsey R. Thomas

**Affiliations:** ^1^ Memory and Aging Center, University of California San Francisco, San Francisco, CA, USA; ^2^ University of California, San Diego, La Jolla, CA, USA; ^3^ San Diego State University/University of California San Diego Joint Doctoral Program in Clinical Psychology, San Diego, CA, USA; ^4^ Johns Hopkins Bloomberg School of Public Health, Baltimore, MD, USA; ^5^ Laboratory of Behavioral Neuroscience, National Institute on Aging, Intramural Research Program, Baltimore, MD, USA; ^6^ Banner Alzheimer's Institute, Tucson, AZ, USA; ^7^ VA San Diego Healthcare System, San Diego, CA, USA; ^8^ University of California San Diego, La Jolla, CA, USA; ^9^ Memory Impairment and Neurodegenerative Dementia Center, University of Mississippi Medical Center, Jackson, MS, USA; ^10^ Optimal Aging Institute, New York University Grossman School of Medicine, New York, NY, USA; ^11^ Mayo Clinic, Rochester, MN, USA; ^12^ National Institute of Neurological Disorders & Stroke, Bethesda, MD, USA; ^13^ University of North Carolina at Chapel Hill, Chapel Hill, NC, USA

## Abstract

**Background:**

Even among cognitively unimpaired (CU) older adults, specific patterns of neuropsychological performance impart differential risk for progression to Alzheimer's disease and related dementias (ADRD). Early identification in the prodromal phase may inform early intervention efforts. We used a data‐driven approach to classify neuropsychological phenotypes within CU older adults from the Atherosclerosis Risk in Communities (ARIC) study and tested for differences in ADRD and cerebrovascular outcomes.

**Method:**

Participants included 4737 CU (via a previously published algorithm plus consensus diagnosis protocol) older adults (mean age=75.1 years, 60.8% female, 23.1% Black) with neuropsychological data from Visit 5 (2011‐2013) of the ARIC study. Latent profile analysis was leveraged to identify unique cognitive phenotypes using 10 demographically adjusted (age, sex/gender, education, race‐center, WRAT Reading) neuropsychological z‐scores. Cognitive phenotypes were then compared on demographics, depressive symptoms, and vascular risk factors (VRFs). In a subset with available neuroimaging and biofluid data (*n* range=1102‐1678), white matter hyperintensity (WMH) and bilateral hippocampal (HV) volumes normalized for intracranial volume and plasma biomarkers (i.e., Aβ42/40, ptau181, NfL, and GFAP) were examined by phenotype. Phenotype comparisons adjusted for demographic variables, VRFs, and an indicator of kidney function (i.e., eGFR‐creatinine) for plasma biomarkers. We applied Tukey adjustment for pairwise comparisons.

**Result:**

A 6‐class solution of cognitive phenotypes emerged as the best fitting model: *Average Overall* (28%), *Low Overall/Very Low Memory* (7%), *Average Overall/Super Memory* (11%), *Subtle Low Overall/Low Executive* (22%), *Subtle Low Executive/High Memory* (13%), and *Average Overall/Low Memory* (19%; Figure 1). Phenotypes differed on education, race, and word reading, despite prior adjustments for these variables when creating the neuropsychological z‐scores (Table 1). Additionally, phenotypes differed on depressive symptoms, VRFs, WMH, HV, Aβ42/40, and ptau181 (all *p*s < .05). The *Subtle Low Overall/Low Executive* group had the highest WMH volumes, the *Subtle Low Executive/High Memory* group had the lowest Aβ42/40, and the *Low Overall/Very Low Memory* group had the lowest HV and highest levels of plasma ptau181 (Table 2).

**Conclusion:**

Cognitive heterogeneity is observed even among CU older adults, with lower executive performance associated with cerebrovascular changes and lower memory associated with certain AD‐related changes, possibly indicating risk for cognitive progression through distinct pathways.